# Delay discounting of rewards and losses, alcohol use, and the influence of socioeconomic factors: A cross‐sectional online study in frequent drinkers

**DOI:** 10.1111/acer.15469

**Published:** 2024-10-22

**Authors:** Mathieu Pinger, Malin Skirke, Janine Thome, Wolfgang H. Sommer, Georgia Koppe, Peter Kirsch

**Affiliations:** ^1^ Institute of Psychology University of Heidelberg Heidelberg Germany; ^2^ Department of Clinical Psychology, Central Institute of Mental Health Mannheim, Medical Faculty Mannheim University of Heidelberg Heidelberg Germany; ^3^ Department of Theoretical Neuroscience, Central Institute of Mental Health Mannheim, Medical Faculty Mannheim University of Heidelberg Heidelberg Germany; ^4^ Department of Psychiatry and Psychotherapy, Central Institute of Mental Health Mannheim, Medical Faculty Mannheim University of Heidelberg Heidelberg Germany; ^5^ Department of Psychopharmacology, Central Institute of Mental Health Mannheim, Medical Faculty Mannheim University of Heidelberg Heidelberg Germany; ^6^ Bethanien Hospital for Psychiatry Psychosomatics and Psychotherapy Greifswald Germany; ^7^ Hector Institute for AI in Psychiatry, Central Institute of Mental Health Mannheim, Medical Faculty Mannheim University of Heidelberg Heidelberg Germany; ^8^ Interdisciplinary Center for Scientific Computing, Faculty for Mathematics and Computer Science University of Heidelberg Heidelberg Germany

**Keywords:** addiction, alcohol use, delay discounting, impulsivity, socioeconomic status

## Abstract

**Background:**

Delay discounting describes the devaluation of future outcomes over time and is a popular behavioral construct in addiction research. Prior studies show modest yet consistent associations between problematic alcohol use and delayed reward discounting (DRD). However, the potential confounding influence of socioeconomic status (SES, e.g., income and education) is rarely addressed. In this study, we aimed to investigate the robustness of DRD as a predictor of alcohol use after controlling for socioeconomic and demographic variables. Additionally, we aimed to test the association between delayed loss discounting (DLD) and alcohol use in a sufficiently large sample.

**Methods:**

We collected data from 341 moderate‐to‐heavy‐drinking participants (27.92 ± 21.12 g alcohol/day, 43.48 ± 11.90 years old, 49.9% female, UK residents) in a cross‐sectional online study. DRD and DLD were measured using an intertemporal choice task. Questionnaires encompassed alcohol use (AUDIT, weekly alcohol consumption), education and income, subjective measures of past and present socioeconomic status, and impulsivity.

**Results:**

DRD, but not DLD, was significantly associated with AUDIT scores (*r* = 0.15) and weekly alcohol consumption (*r* = 0.12). DRD remained a significant yet weak predictor of AUDIT scores when controlling for education and income, but not when controlling for education and age.

**Conclusions:**

We replicated a small but robust association between alcohol use and DRD, but not DLD. This association appeared to be confounded by education and age, but not by income. We conclude that socioeconomic and demographic variables should systematically be accounted for in future studies investigating DRD and alcohol use.

## Graphical abstract

This study investigated the link between delayed reward (DRD), delayed loss discounting (DLD), and alcohol use, considering socioeconomic status (SES) and demographic factors. In 341 moderate‐to‐heavy‐drinking UK adults, alcohol use was associated with DRD, but not DLD. The association between DRD and alcohol use persisted when controlling for education and income, but not education and age. These findings highlight the importance of accounting for SES and demographic variables in addiction research.

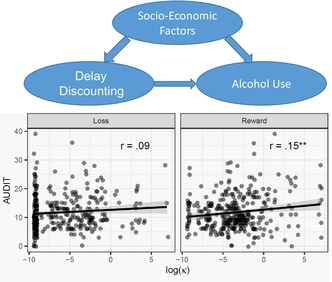

## INTRODUCTION

### Reward discounting, addiction, and socioeconomic status

Numerous studies have established a link between delayed reward discounting (DRD) and alcohol use disorder (AUD), demonstrating that individuals with more AUD symptoms and higher drinking levels tend to favor short‐term rewards in monetary intertemporal choice tasks (Amlung et al., 2017; MacKillop et al., 2011). Excessive DRD is often interpreted as a marker of aberrant and impulsive decision making serving both as a potential risk factor and a consequence of addiction and thus a potential target for prevention and therapy (Bickel et al., 2014; Story et al., 2014).

However, a growing body of research suggests that DRD reflects more than impulsivity alone. Apart from a general debate on the construct of impulsivity and its relation to DRD (for an in‐depth discussion, see Stahl et al., 2014; Strickland & Johnson, 2021), steep DRD can be construed as a rational behavior in response to limited resources when waiting for a delayed reward is not feasible (Becker & Mulligan, 1997; Ruggeri et al., 2022). Indeed, lower income is consistently associated with steeper DRD, which is unsurprising given the inherent financial nature of most DRD tasks (de Wit et al., 2007; Green et al., 1996, 2014; Reimers et al., 2009). Findings that hypothetical income declines are associated with increased DRD rates seem to suggest a causal link between income and DRD rates (Bickel et al., 2016; Mellis et al., 2018). Recently, global analyses of financial decision making indicated steeper DRD rates in low‐income populations, but no effect of DRD on upward economic mobility, suggesting a causal influence of economic inequality on DRD, but not vice versa (Ruggeri et al., 2022, 2023). In addition to income, other indices of socioeconomic status (SES) across the lifespan have been related to DRD rates, with education being the most prominent example (Amlung & MacKillop, 2014; de Wit et al., 2007; Ishii et al., 2017; Jaroni et al., 2004; Reimers et al., 2009). Some associations between SES during adolescence (e.g., parental education and income) and DRD rates seem to suggest a causal effect of SES on DRD (Anokhin et al., 2011; Tunney, 2022).

Individuals with low SES not only discount rewards more steeply but also have a higher risk of AUD and mental disorders (Beard et al., 2019; Grant et al., 2015; Jenkins et al., 2008). Moreover, their risk for alcohol‐related harm is disproportionally higher when compared to individuals with higher SES and equal drinking levels (Shuai et al., 2022). In summary, experiencing socioeconomic hardship increases both DRD rates and the vulnerability of AUD. Despite this, SES such as income and education are not systematically considered as potential confounder variables in studies investigating the association between DRD and alcohol use. One reason for this could be that many studies are limited to relatively small clinical populations that have already experienced significant socioeconomic consequences due to AUD. Considering the small effects of DRD typically observed in addiction studies (Amlung et al., 2017), reliably disentangling true and confounding effects requires large samples with sufficient variance. Notably, correcting for education and income has been shown to weaken associations between DRD (and the closely related concept of delay‐of‐gratification) and outcomes related to addiction and health (Acheson et al., 2019; Amlung & MacKillop, 2014), but also working memory, cortical volume, and future life achievements (Garzón et al., 2022; Watts et al., 2018). Therefore, we set out to test the robustness of DRD as a predictor of alcohol use when controlling for SES in a relatively large sample with a broad distribution of alcohol use.

### Delayed loss discounting and AUD

Decisions about alcohol consumption involve assessing both positive and negative consequences. High‐risk drinkers may not only devaluate the benefits of abstinence, but also the long‐term harms of drinking, leading to a choice bias towards larger‐later aversive consequences. However, research on alcohol use and delayed loss discounting (DLD) is scarce. In a study of 33 students, DLD was moderately linked to alcohol consumption frequency (Takahashi et al., 2009). Bailey et al. (2018) and Gerst et al. (2017) found that individuals with AUD discount future losses more than healthy controls. Those who steeply discount rewards also tend to discount losses more steeply (DeHart et al., 2020; Thome, Pinger, Halli, et al., 2022). However, Myerson et al. (2017) found significant associations between AUD and DRD, but not DLD. Interestingly, in a prior exploratory analysis in a healthy sample, we observed the opposite pattern (Thome, Pinger, Halli, et al., 2022). Evidence from larger samples with problematic alcohol use is lacking, and therefore DLD remains an under‐researched candidate paradigm for AUD research.

### Aims and hypotheses

The study pursued a combination of confirmatory hypothesis testing and exploratory data analysis. For the confirmatory part, we aimed to address two key questions related to DRD and its association with alcohol use. First, we sought to investigate to which degree the relationship between DRD and alcohol use is confounded by SES. We selected income and education as primary SES variables due to the consistent literature suggesting an influence of these variables on DRD and alcohol use. Second, we aimed to investigate a potential relationship between alcohol use and the discounting of monetary losses. Hypotheses and analysis strategies for the confirmatory part were preregistered (https://aspredicted.org/ac46k.pdf). We hypothesized to replicate the positive association between alcohol use (measured by AUDIT scores) and DRD steepness (H1) and extend this to DLD steepness (H2). Furthermore, we hypothesized that SES (yearly income and education) is negatively associated with DRD (H3) and AUD severity (H4). Lastly, we hypothesized that controlling for these SES indices diminishes the association between DRD and alcohol use (H5).

For the exploratory part, we collected a number of additional variables including demographic data, different indices of delay discounting, and a range of further socioeconomic variables of potential interest.

## MATERIALS AND METHODS

### Sample

Participants used personal computers at home and were recruited in June 2022 via the online participant platform Prolific (https://www.prolific.co). Eligibility criteria were filtered using Prolific's custom prescreening tools and included age 18–65, current residency in the UK, and minimum weekly alcohol consumption of 10 alcohol units. To enhance data quality, we restricted eligibility to participants who had previously completed at least five Prolific studies with an approval rating of 95% or higher. Participation in our previous delay discounting studies was an exclusion criterion. Sampling was balanced with respect to gender. Participants received £9 per hour as compensation and provided informed consent prior to the study. The ethics committee of the Medical Faculty Mannheim, University of Heidelberg (2019‐633°N), approved the study.

To maximize variance with respect to both SES and alcohol use, we pursued a varied nonclinical sample of individuals with moderate to heavy drinking patterns. This allowed us to test our main hypotheses on a sample of participants ranging from occasional to highly risky drinking behavior. A priori power analysis determined a required sample size of 311 participants for 80% power to replicate the correlation of *r* = 0.14 (two‐sided) between DRD and alcohol use found in a meta‐analysis (Amlung et al., 2017). We targeted a sample size of 350 participants to reliably detect a comparable correlation between DLD and alcohol use. In addition, prior studies indicated correlations between SES and both DRD and alcohol use in a comparable or higher effect size range (Amlung & MacKillop, 2014; Ishii, 2015; Ishii et al., 2017; Najdzionek et al., 2023).

### Study materials

#### Delay discounting of rewards and losses

Participants completed an intertemporal choice task developed by our group (Thome, Pinger, Halli, et al., 2022), making decisions between hypothetical monetary rewards (reward condition, 96 trials) or losses (loss condition, 96 trials). Each choice involved an immediate smaller outcome and a delayed larger outcome, with varying delays (*D* = {7, 30, 90, 180, 365, 109} days), immediate reward/loss magnitudes (*r*_a1_), and delayed reward/loss magnitudes (r_a2_ = {5, 10, 20 50} £UK). Immediate magnitudes (*r*_a1_) were determined a priori through a computational model solving for magnitudes for a range of hypothetical discounting parameters and predicted choice probabilities. We could show that this procedure, compared to other fixed‐trial procedures as in Rachlin et al. (1991), samples sufficient variance in behavior across a broad range of plausible discounting rates (for details, see Thome, Pinger, Durstewitz, et al., 2022, Thome, Pinger, Halli, et al., 2022).

The 96 trials of each condition were randomized and split into two blocks of 48 trials each.

Reward and loss blocks were presented in alternating order, starting randomly with either condition. Within each trial, the two options were randomly presented on the left and the right side of the screen. Participants indicated their choices by pressing either “Q” (for the left option) or “P” (for the right option) within 10 s of the stimulus presentation. The chosen option was then highlighted for 1 s, followed by a fixation cross for another second. After each block, participants were allowed to take a break for a self‐chosen duration.

#### Self‐report measures

Details on all self‐report measures, including wording and response options for each question and item, are provided in the online codebook (https://osf.io/85k3h/).

Problematic alcohol use was assessed using the Alcohol Use Disorder Identification Test (AUDIT, Saunders et al., 1993). To enhance standardization, the term “a drink” was replaced with “a standard unit,” accompanied by a visual aid retrieved from the UK Department of Health and Social Care (2020). Using the Daily Drinking Questionnaire (DDQ; Collins et al., 1985), participants reported average standard units of alcohol consumed on each day of the week over the past 3 months.

Assessment of income was based on gross income in the last 12 months, including earnings from all sources of income, separately for individual and household levels. Income was assessed using levels from “less than £10,000” to “more than £250,000,” using £10,000 increments up to £100,000, and thereafter in £50,000 increments (Diemer et al., 2013). Education was assessed as the highest level of education according to the International Standard Classification of Education (ISCED) levels adapted for the UK (Schneider, 2013), ranging from 0 (“no formal qualification”) to 7 (“doctoral degree or higher”). Education of primary and secondary (if applicable) caregivers during adolescence was assessed using the same levels. According to some studies, subjective measures of SES may relate more strongly to alcohol use and psychological well‐being compared to objective measures (Garza et al., 2017; Ishii, 2015; Ishii et al., 2017; Najdzionek et al., 2023). Therefore, we also measured subjective SES using the MacArthur scale (Adler et al., 2000). The scale asks participants to rate their relative subjective socioeconomic well‐being including finances, occupation, and education from 1 (worst off, least money, least education, worst job) to 10 (best off, most money, best education, best job). Subjective financial well‐being during adolescence was measured using the single‐item question “Please rate your family's or household's financial wellbeing during your adolescence” and a Likert scale with five steps (“not at all well‐off,” “not very well‐off,” “average,” “somewhat well‐off,” and “very well‐off”).

Lastly, to provide additional data on the discussion surrounding DRD versus impulse personality traits, impulsivity was measured using the short‐form Barratt Impulsiveness Scale (BIS‐15; Spinella, 2007).

#### Data collection and study procedure

The online study was programmed in JavaScript using the open‐source package jsPsych, version 6.2 (de Leeuw, 2015) and was hosted on a custom virtual server using a Linux‐Apache‐MySQL‐PHP stack (see Thome, Pinger, Durstewitz, et al., 2022, Thome, Pinger, Halli, et al., 2022 for details; note that only “run A” was executed to estimate discounting parameters). Participants entered the study through a link on the Prolific website. After completing the consent form and filling out sociodemographic information, participants received an introduction to the intertemporal choice tasks, including six example trials. After finishing the task, participants completed the remaining questionnaires.

#### Data analysis

##### Data preprocessing

Data from participants who completed less than 80% of the discounting trials within one condition, who displayed stereotypical key press patterns (only pressing “Q” or “P” despite randomly changing locations of the immediate/delayed options), or who had average reaction times below 500 ms in the discounting trials were excluded from all analyses. Data exclusion was preregistered.

Sum scores were calculated for the AUDIT, the BIS‐15, and the DDQ. Education and income were treated as continuous variables. To this end, the character‐based income levels (e.g., “£10,000–20,000”) were transformed into numerical values using midpoints of each income category (£15,000 for the example above). The ISCED education categories were treated as a Likert scale. Educational levels of primary and secondary caregivers during adolescence were averaged to obtain single‐value parental education levels.

Intertemporal decision making was investigated using discounting frequencies and hyperboloid model parameters. To this end, hyperboloid discounting models were inferred from the behavioral choices of each participant (see Thome, Pinger, Durstewitz, et al., 2022; Thome, Pinger, Halli, et al., 2022 for details). The modified hyperboloid model (Mazur, 1987; Rachlin, 2006) posits that the values *V* for the delayed options *a*_2_ are discounted according to

(1) $$V\left(a_{2}\right)=\frac{1}{1+\kappa \cdot D^{s}}r_{2}$$

while the values for the immediate options $a_{1}$ correspond to the actual outcomes, $V\left(a_{1}\right)=r_{1}$. Here, κ indexes the individual discounting parameter, s represents an individual temporal scaling parameter, *D* the temporal delay in days, and $r_{1}\;\text{and}\;\;r_{2}$ are the immediate and delayed outcomes, respectively. Values were translated into immediate choice probabilities via a sigmoid function:

(2) $$p\left(a_{1}|V\right)=\frac{1}{1+e^{\beta \left(V\left(a_{2}\right)-V\left(a_{1}\right)\right)}},$$

where β indicates the disposition to exploit (β→∞) or explore (β→0) choices (Sutton & Barton, 2018), and $p\left(a_{2})=1-p(a_{1}\right)$. Parameters were then inferred via maximum likelihood estimation (see also Ahn et al., 2020; Thome, Pinger, Durstewitz, et al., 2022; Thome, Pinger, Halli, et al., 2022), implemented via optimize.minimize() from the SciPy library in Python, with constraints κ ϵ [0, 1000] and β ϵ [0.01, 2]). Separate discounting and scaling parameters were inferred for DRD and DLD trials.

Lastly, κ parameters were obtained from the hyperboloid model (see above) and underwent natural log transformation after adding a constant of 0.0001 to account for zero values. Log(κ) is the most common index of discounting and determines the steepness of the devaluation of future rewards/losses according to a hyperboloid curve. Higher log(κ) values indicate that steeper discounting Log(κ_R_) is used to refer to DRD, and log(κ_L_) is used to refer to DLD.

Two exploratory indices of discounting behavior (relative frequency of discounted choices, discounting factor at one‐year delay) were also extracted to test the robustness of the main results beyond hyperboloid model parameters. Details are described in Data S1.

##### Hypothesis testing

All data were analyzed using R, Version 4.2.1 (R Core Team, 2022). Outputs for multiple regression were generated using the package *apaTables*, version 2.0.8 (Stanley, 2015), which calculates confidence intervals for Δ*R* ^2^ and semi‐partial correlations using the Alf Jr. and Graf (1999) method. Hypothesis testing was two‐tailed using α = 0.05 and followed the preregistered analysis plan.

The predictive effects of DRD (H1) and DLD (H2) on problematic alcohol use were assessed through simple linear regressions with AUDIT sum scores as the dependent variable and log(κ_R_) for DRD or log(κ_L_) for DLD as the independent variables. Predictive effects of SES on DRD (H3) and problematic alcohol use (H4) were tested through multiple linear regressions, using education and individual income as independent variables and log(κ_R_) and AUDIT scores as dependent variables, respectively. Confounding effects of SES on the association between DRD and problematic alcohol use (H5) were tested through hierarchical linear regression. Individual income and education were introduced as independent variables, followed by log(κ_R_) in a subsequent step. F‐tests were used to test whether the addition of log(κ_R_) explained significantly more variance than SES alone.

##### Exploratory analyses

Pearson correlation coefficients were obtained for pairwise combinations of all variables.

Regression models for hypothesis testing were repeated for the two exploratory measures of delay discounting and further variables significantly associated with both delay discounting and alcohol use.

We examined gender effects via *t*‐tests on AUDIT scores, DDQ scores, discounting, impulsivity, education, and income. Additionally, we employed a multiple regression model with log(κR), gender (dummy‐coded with 0 = female, 1 = male) and their interaction as independent variables, and AUDIT scores as the dependent variable.

Participants with a relative discounting frequency below 5% in the intertemporal choice tasks were defined as nondiscounters. *T*‐tests were conducted to compare discounters and nondiscounters with respect to age, impulsivity, alcohol use, income, and education.

## RESULTS

### Sample description and missing data

**FIGURE 1:**
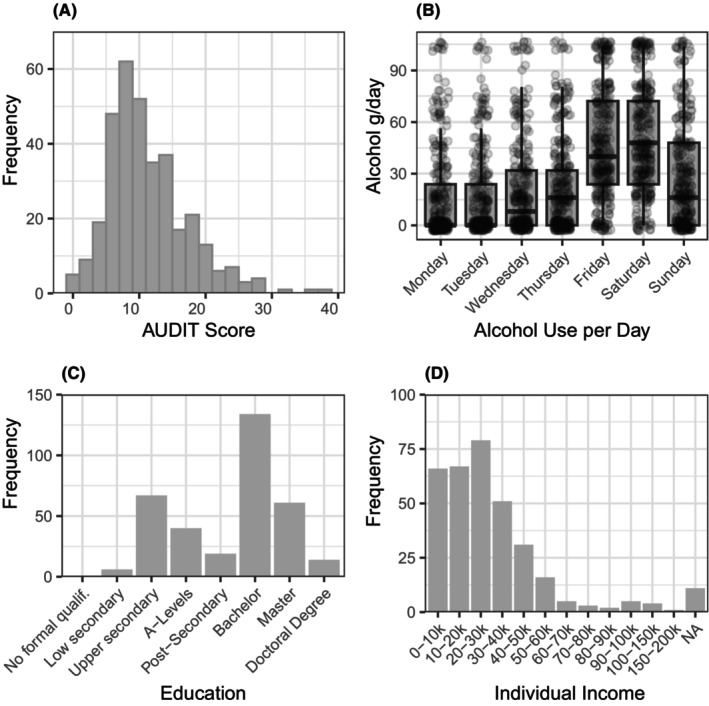
Distribution of alcohol‐related and socioeconomic variables. (A) Histogram of AUDIT sum scores. (B) Average daily drinking quantities over the course of the last 3 months as assessed by the Daily Drinking Questionnaire (DDQ). For easier interpretation, UK alcohol units (= 8 g) were transformed into grams per day. (C) Education levels according to ISCED. Details on the educational levels can be found in the supplementary codebook. (D) Distribution of individual income.

A total of 347 participants completed the online study. Due to technical problems, data from six participants could not be retrieved from the server; therefore, the final sample size is *N* = 341. Tables 1 and 2 provide descriptive statistics of all variables including missing values. On average, participants had an AUDIT score of 11.76 and had been drinking 24.43 alcohol units per week or 27.92 g/day of alcohol within the 3 months precluding the study. Distributions of AUDIT scores, education and income levels, and daily alcohol consumption, are illustrated in Figure 1. No participants had to be excluded based on reaction times or key press patterns in the delay discounting tasks. One participant exceeded the exclusion criteria of more than 20% missing trials in the loss condition. Due to a few missing data entries, participants with missing data points were excluded from analyses with the missing variable.

**TABLE 1:** Descriptive statistics (numeric).

Variable	*N*	Mean	SD	SE	Min	Max	Skew	Kurtosis
Age	341	43.48	11.90	0.64	19.00	65.00	−0.19	−0.89
Education	341	4.31	1.57	0.08	1.00	7.00	−0.40	−1.04
AUDIT	341	11.76	6.06	0.33	0.00	39.00	1.04	1.70
DDQ (8 g Alc. units per week)	341	24.43	18.48	1.00	0.00	91.00	1.50	2.28
Income individual (£)	330	28,000.00	22,846.79	1257.67	5000.00	175,000.00	2.24	8.11
Income household (£)	329	53,541.03	39,586.26	2182.46	5000.00	250,000.00	2.09	6.48
No. of household members	341	2.60	1.25	0.07	1.00	7.00	0.88	0.72
Subjective SES	341	5.53	1.63	0.09	1.00	10.00	−0.33	−0.31
Adolescent well‐being	341	1.77	0.93	0.05	0.00	4.00	0.11	−0.32
Avg. parental education	318	2.43	1.73	0.10	0.00	7.00	0.55	−0.63
BIS‐15	341	30.65	6.65	0.36	16.00	52.00	0.30	0.02
Reward discounting								
Rel. discounting frequency	341	46.88	18.54	1.00	0.00	100.00	−0.17	0.07
β	341	0.89	0.68	0.04	0.00	2.00	0.52	−1.17
*s*	341	0.49	0.37	0.02	0.00	1.00	0.02	−1.48
log(κ_R_)	341	−3.44	3.40	0.18	−9.21	6.91	0.63	0.81
Discounting factor	341	0.57	0.31	0.02	0.00	1.00	−0.38	−1.10
Loss discounting								
Rel. discounting frequency	340	25.08	22.20	1.20	0.00	100.00	0.53	−0.61
β	340	1.11	0.78	0.04	0.00	2.00	0.04	−1.73
*s*	340	0.30	0.38	0.02	0.00	1.00	0.83	−0.91
log(κ_L_)	340	−5.99	3.77	0.20	−9.21	6.91	1.07	0.54
Discounting factor	340	0.77	0.30	0.02	0.00	1.00	−1.24	0.34

*Note*: β = exploitation–exploration parameter. s = exponential temporal scaling parameter of hyperboloid model. log(κ_R_) and log(κ_L_) = natural log‐transformed discounting parameter of the hyperboloid model. For details on model parameters, see Thome, Pinger, Halli, et al. (2022) and Data S1.

Abbreviations: AUDIT, Alcohol Use Disorders Identification Test; BIS‐15, Barratt Impulsiveness Scale (Short Version); DDQ, Daily Drinking Questionnaire; SD, standard deviation, SE, mean standard error, SES, socioeconomic status.

**TABLE 2:** Descriptive statistics (categorical).

Variable	Categories	*N*	%
Gender	Female	170	49.9
	Male	171	50.1
Employment	Employed	224	65.7
	Self‐employed	50	14.7
	Retired	15	4.4
	Student	14	4.1
	Unemployed	25	7.3
	Other	13	3.8

### Preregistered hypotheses

Pearson correlation coefficients between the five variables preregistered for hypothesis testing are given in Table 3.

**TABLE 3:** Means, standard deviations, and correlations with confidence intervals for the variables used in preregistered hypothesis testing.

Variable	AUDIT	Log(κ)—reward	Log(κ)—Loss	Individual income
AUDIT				
log(κ)—Reward	0.15** [0.04, 0.25]			
log(κ)—Loss	0.09 [−0.02, 0.19]	0.25** [0.15, 0.35]		
Individual Income	−0.12* [−0.22, −0.01]	−0.10 [−0.20, 0.01]	0.00 [−0.10, 0.11]	
Education	−0.15** [−0.25, −0.05]	−0.19** [−0.29, −0.08]	−0.07 [−0.17, 0.04]	0.26** [0.16, 0.36]

*Note*: For the purpose of legibility, the complete table of all pairwise correlations has been moved to Data S2. Values in square brackets indicate the 95% confidence interval for each correlation. *p < 0.05. **p < 0.01.

**FIGURE 2:**
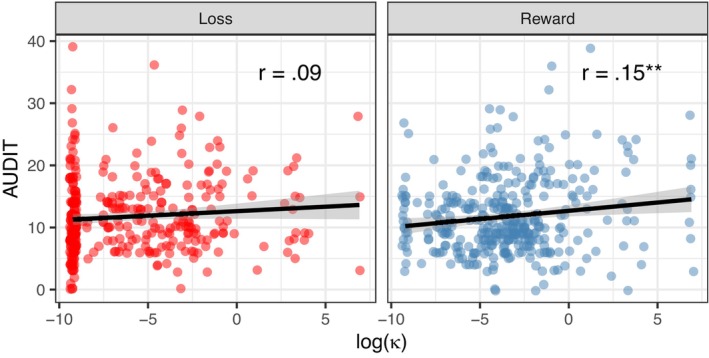
Associations between hyperboloid discounting parameters log(κ) and AUDIT sum scores in the reward (*N* = 341) and loss (*N* = 340) condition. Higher log(κ) indicates steeper discounting. *p < 0.05, **p < 0.01.

*H1: DRD and Problematic Alcohol Use*. We found a small but significant positive correlation between the DRD parameter log(κ_R_) and AUDIT sum scores (*r* = 0.15, *p* = 0.01, Figure 2). Linear regression revealed a significant prediction of AUDIT scores by log(κ_R_) (*R* ^2^ = 0.023, *F*(1, 339) = 7.81, *p* < 0.01).

*H2: DLD and Problematic Alcohol Use*. The correlation between the DLD parameter log(κ_L_) and AUDIT sum scores was not significant (*r* = 0.09, *p* = 0.10, Figure 2); therefore, no linear regression was computed.

*H3: DRD and SES*. We found a significant negative correlation between the DRD parameter log(κ_R_) and level of education (*r* = −0.19, *p* < 0.01), but not between log(κ_R_) and yearly individual income (*r* = −0.10, *p* = 0.07). Linear regression revealed a significant prediction of log(κ_R_) by education (*R* ^2^ = 0.031, *F*(1, 339) = 12.04, *p* < 0.01).

*H4: SES and Problematic Alcohol Use*. AUDIT sum scores were significantly and negatively correlated with both level of education (*r* = −0.15, *p* < 0.01) and yearly individual income (*r* = −0.12, *p* = 0.03), with the latter two also showing a significant intercorrelation (*r* = 0.26, *p* < 0.01). Employing both education and income as independent variables and AUDIT scores as dependent variables in multiple regression, only education remained a significant predictor (β = −0.11, *t*(327) = −2.03, *p* = 0.04). The fitted model accounted for 2.6% of the variance in AUDIT scores (*R* ^2^ = 0.026, *F*(2, 327) = 4.43, *p* = 0.01, see Table 4).

**TABLE 4:** Hierarchical regression results using AUDIT sum scores as the criterion.

Predictor	*b*	*b*, 95% CI [LL, UL]	*β*	*β*, 95% CI [LL, UL]	*sr* ^*2*^	*sr* ^*2*^, 95% CI [LL, UL]	*r*	Fit	Difference
*Step 1*									
(Intercept)	14.45**	[12.51, 16.40]							
Education	−0.45*	[−0.88, −0.01]	−0.11	[−0.23, −0.00]	0.01	[−0.01, 0.04]	−0.14*		
Individual Income	−0.00	[−0.00, 0.00]	−0.09	[−0.20, 0.02]	0.01	[−0.01, 0.03]	−0.12*		
								*R* ^*2*^ = 0.026*	
								95% CI [0.00, 0.07]	
*Step 2*									
(Intercept)	14.79**	[12.83, 16.74]							
Education	−0.36	[−0.80, 0.08]	−0.09	[−0.20, 0.02]	0.01	[−0.01, 0.03]	−0.14*		
Individual Income	−0.00	[−0.00, 0.00]	−0.08	[−0.19, 0.03]	0.01	[−0.01, 0.02]	−0.12*		
log(κR)	0.22*	[0.03, 0.42]	0.13	[0.02, 0.23]	0.02	[−0.01, 0.04]	0.15**		
								*R* ^*2*^ = 0.042**	Δ*R* ^*2*^ = 0.015*
								95% CI [0.01, 0.08]	95% CI [−0.01, 0.04]

*Note*: *b* represents unstandardized regression weights. *β* indicates the standardized regression weights. *sr* ^*2*^ represents the semi‐partial correlation squared. *r* represents the zero‐order correlation. *LL* and *UL* indicate the lower and upper limits of a confidence interval, respectively.

*p < 0.05; **p < 0.01.

*H5: DRD, SES, and Problematic Alcohol Use*. When the DRD parameter log(κ_R_) was included as an independent variable after testing education and income alone, the extended model explained significantly more variance in AUDIT sum scores than the simpler model (Δ*R* ^2^ = 0.015, Total *R* ^2^ = 0.041, *F*(3, 326 = 5.16), *p* = 0.02, see Table 4). However, the 95% confidence interval for this increase in *R* ^2^ included zero (95% CI [−0.01, 0.04]), suggesting that the increase in explained variance is marginal. In addition, log(κ_R_) remained the only significant predictor of AUDIT scores (β = 0.13, *t*(326) = 2.27, *p* = 0.02).

### Exploratory analyses

Pairwise correlations between all measures of alcohol consumption, delay discounting, SES, and demographic variables are given in Data S2.

### Exploratory measures of SES

Among exploratory measures of current and past SES, only current subjective SES was found to be significantly associated with AUDIT scores (*r* = 0.14) and log(κ_R_) (*r* = 0.19, Data S2). Rerunning the hierarchical regression used for H4 and H5 with subjective SES and education as first‐level independent variables revealed that log(κ_R_) remained a significant predictor of AUDIT scores (Data S3.3).

### Exploratory measures of alcohol use and discounting

All three indices of DRD (log(κ_R_), relative frequency of discounted choices, discounting factor at 1‐year delay) were highly intercorrelated, as well as the two measures of alcohol use (AUDIT, DDQ, see Data S2). Therefore, the regression models used to test H4 and H5 were repeated for these secondary measures. Results are provided in Data S3.1, S3.2, and S3.4. All measures of DRD remained significant predictors of alcohol use (AUDIT and drinking quantity) when controlling for SES.

### Loss discounting

The three indices of DLD (log(κ_L_), the relative frequency of discounted choices, discounting factor at 1‐year delay) were highly intercorrelated (see Data S2). Small but significant correlations were present between DRD and DLD (e.g., *r* = 0.25 between log(κ_R_) and log(κ_L_)). In contrast to DRD, no measure of DLD was significantly correlated with any of the socioeconomic or alcohol‐related variables.

On average, DLD was less steep than DRD, as indicated by lower frequencies of discounting behavior (46.88% in DRD vs. 25.08% in DLD). Importantly, 29.7% of participants were nondiscounters in the DLD condition, compared to only 2.9% of participants in the DRD condition. Paired *t*‐tests revealed that nonDLD‐discounters did not differ from other participants in age, alcohol use, impulsivity, education, and income (Data S4). Lastly, when we excluded nondiscounters from the correlation analysis for H2 to rule out a possible subgroup effect, the association between log(κ_L_) and AUDIT sum scores remained nonsignificant (*r* = 0.07, *p* = 0.31).

### Demographic variables

In addition to SES, we found significant correlations between alcohol use, DRD, and age. Age was negatively correlated with log(κ_R_) (*r* = −0.20, *p* < 0.01) and AUDIT scores (*r* = −0.22, *p* < 0.01). When age and education were entered as first‐step predictors in a hierarchical regression similar to H4 and H5, 7.1% of the variance in AUDIT scores could be explained (*F*(2, 338) = 13, *p* < 0.01, Data S3.5), with both predictors reaching significance. The addition of log(κ_R_) accounted for an additional 0.7% of the variance but did not reach statistical significance (*F*(1,337) = 2.40, *p* = 0.12).

**FIGURE 3:**
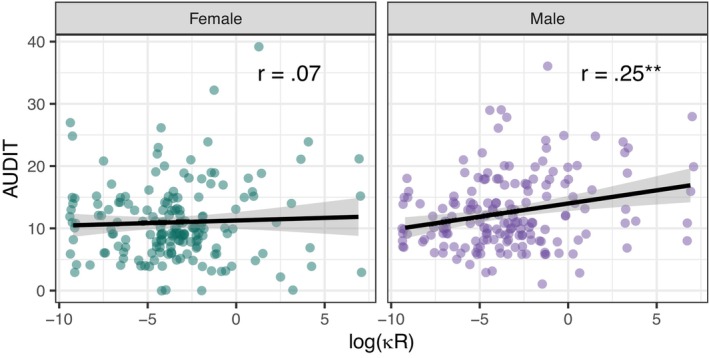
Associations between hyperboloid discounting parameters log(κ_R_) and AUDIT sum scores in the male (*N* = 171) and female (*N* = 170) subgroups. Higher log(κ_R_) indicates steeper reward discounting. *p < 0.05, **p < 0.01.

Women and men did not differ significantly with respect to measures of DRD and DLD. On average, women had significantly lower AUDIT scores, drinking quantities, and individual (but not household) income (Data S5). Upon visual inspection, the association between log(κ_R_) and AUDIT scores appeared higher in men (*r* = 0.25, *p* < 0.01) than in women (*r* = 0.05, *p* = 0.55) (Figure 3). However, the difference in correlation coefficients was not significant when applying Fisher's *z*‐transformation (*z* = 1.91, *p* = 0.06). Multiple regression revealed a significant main effect of gender (*t* = 2.98, *p* < 0.01), but no main effect of log(κ_R_) (*t* = 0.61, *p* = 0.54) and no significant interaction effect of gender and log(κ_R_) (*t* = 1.79, *p* = 0.07; Data S3.6) on AUDIT scores. Regarding log(κ_L_), neither the correlation within the male (*r* = 0.14, *p* = 0.08) nor in the female subsample (*r* = 0.06, *p* = 0.47) reached significance, and there was no significant difference in correlation coefficients (*z* = 0.73, *p* = 0.46).

### Impulsivity

BIS‐15 sum scores were positively correlated with log(κ_R_) (*r* = 0.17, *p* < 0.01) and AUDIT scores (*r* = 0.36, *p* < 0.01). When employing both BIS‐15 scores and log(κ_R_) as independent variables in a multiple regression with AUDIT scores as the dependent variable, only BIS‐15 scores explained significant variance *R* ^2^ = 0.13, *F*(2, 338) = 27.32, *p* < 0.01, Data S3.7).

## DISCUSSION

Our study sought to elucidate the complex relationship between delay discounting, socioeconomic status (SES), and the severity of problematic alcohol use. Namely, we investigated (1) the confounding role of SES on the association between delayed reward discounting (DRD) and problematic alcohol use, and (2) the relationship between delayed loss discounting (DLD) and problematic alcohol use. Our findings revealed that DRD remained a weak yet significant predictor of problematic alcohol use when controlling for SES only (income and education), but not when controlling for education and age. On the other hand, DLD rates were not significantly associated with alcohol use.

### DRD, problematic alcohol use, and SES

Consistent with our hypotheses and previous research, our findings revealed significant associations between DRD, alcohol use, and SES. The small yet significant positive correlations between log(κ_R_) and AUDIT (*r* = 0.15) and weekly drinking quantity (*r* = 0.12) replicate the meta‐analytic effects of *r* = 0.14 between DRD and AUDIT and *r* = 0.11 between DRD and drinking quantity (Amlung et al., 2017). In addressing our first research question, we discovered that DRD significantly contributes to explaining alcohol use, even after accounting for education and income (Table 4). This finding remained robust in exploratory models using various measures of DRD and alcohol use, and accounting for subjective SES (Data S3.1–S3.4). Notably, the effect size of DRD after controlling for SES is very small, explaining only 1.1%–1.5% of the variance in AUDIT scores, depending on the model. While DRD *b* weights and ΔR^2^ were statistically significant, the confidence intervals for Δ*R* ^2^ consistently included zero (Table 4, Data S3.1–S3.4). This discrepancy can occur with small effect sizes and multicollinearity but also depends on the exact method of confidence interval estimation (Cumming & Finch, 2005). Taken together, the significance of Δ*R* ^2^ at *p* = 0.02 alongside a 95% CI that includes zero suggests that the result is marginal. The evidence suggests that DRD may have a limited unique contribution to AUDIT scores or that a larger sample is required to accurately estimate its effect when accounting for confounders. Even adjusting for education alone produced this effect, which may explain why DRD research often overlooks confounding variables.

Contrary to prior findings (Amlung & MacKillop, 2014), neither individual nor household income was significantly related to DRD. In addition, education emerged as a stronger predictor of alcohol use than income, which replicates the main finding from a large UK‐based survey (Beard et al., 2019). Given the face validity of financial background influencing monetary decision making, the lack of income effects surprised us. The use of an online sample of paid participants could be a biasing factor here, as financial reward is possibly a relevant motivational factor for Prolific participants. However, the observed mean income of £28.000 matches the median UK income of £27.200 in 2022, and the distribution of income (Figure 1D) resembles UK population data (HM Revenue and Customs, 2022). An important variance‐limiting factor is our decision to use broad income categories of £10.000 each that should possibly be set smaller in future investigations. However, not all prior studies reported an effect of income on DRD rates (e.g., Mitchell et al., 2005), therefore it is well possible that income is a weaker driver of DRD than expected.

In contrast to education, adolescent SES and parental education showed no substantial association with alcohol use or DRD. This contrasts with previous findings linking addiction and monetary decision making to socioeconomic hardship in childhood and youth (Hardaway & Cornelius, 2014; Tunney, 2022). However, questions about retrospective subjective SES may be prone to biased memory and do not encompass all dimensions of adolescent SES. Therefore, the role of SES over the lifespan can only be answered in longitudinal designs.

### DRD, problematic alcohol use, and demographic variables

Interestingly, when education and age were accounted for, DRD did not explain significant variance. Older and more educated individuals reported lower problematic alcohol use and discounted rewards less steeply. This effect was found in an exploratory analysis and not expected by us since a recent meta‐analysis did not report any systematic association between DRD and age (Seaman et al., 2020). In addition, age and education can be mutually confounding (18‐year‐old participants are unlikely to have obtained a university degree), although this is only relevant for a small portion of participants. Therefore, while this finding casts doubt on the incremental share of variance explained by DRD, we suggest careful confirmation in a subsequent study. Additionally, unlike income, the effect of age does not necessarily challenge the validity of DRD as a measure in addiction research, as the relationship between age, DRD, and alcohol use could be mediating rather than confounding. In contrast, income and education are directly relevant for health policy decisions, as the question arises as to whether it is primarily individual decision‐making behavior or economic conditions that contribute to the prevention of addiction.

As women are underrepresented in addiction research (Agabio et al., 2017), we aimed at a balanced sample of male and female participants. We found no significant main effects of gender on DRD or DLD, aligning with a recent meta‐analysis which found no substantial difference in discounting behavior between men and women (Doidge et al., 2021). Visual inspection of the data suggested that the relationship between DRD and AUDIT scores may be driven by male individuals, as indicated by a correlation coefficient of *r* = 0.25 for men compared to *r* = 0.05 for women (Figure 3). However, a possible moderation effect of gender could not be statistically confirmed (Data S3.6). Therefore, we suggest reexamining this effect in a study with higher power.

### DLD and problematic alcohol use

Contrary to our expectations (H2), neither the delayed loss discounting (DLD) parameter log(κ_L_) nor the two other measures of DLD showed a significant correlation with problematic alcohol use or drinking quantity. As in our earlier study (Thome, Pinger, Halli, et al., 2022), we observed a large percentage (29.7%) of participants who almost never chose the larger‐later loss, compared to only 2.9% of participants who almost never chose the smaller sooner reward. These nondiscounters did not differ significantly from “regular” discounters in education, income, age, drinking level or impulsivity, and their exclusion did not change the overall result. Taken together, we did not find any evidence that DLD is a relevant predictor of problematic alcohol use. However, this does not rule out a potential link between discounting of aversive consequences in other modalities (such as health). Arguably, negative consequences of alcohol cannot be reduced to monetary losses only, psychologically aversive outcomes such as craving, withdrawal, and health problems may play a greater role. Therefore, using monetary DLD to measure flawed decision making in addiction may not be entirely valid. While the same problem applies to monetary DRD, studies show moderate correlations between various reward discounting forms (e.g., money, health) and both hypothetical and real rewards. DLD lacks such evidence due to ethical or scalability issues in studying aversive consequences.

### Delay discounting in addiction research

Lately, there has been a burgeoning debate regarding the validity of DRD as a construct in addiction research (Bailey et al., 2021; Exum et al., 2023, but see also Martínez‐Loredo, 2023; Stein et al., 2023). Responding to the methodological recommendations brought up in this debate, we employed a novel intertemporal choice task that samples behavior across a sufficiently large variety of decisions, and report several measures of DRD/DLD to prevent spurious effects based on inaccurate behavioral models. The finding that hyperboloid model parameters and behavioral frequencies seem to yield the same results supports the robustness of our findings.

Our hypothesis for this study was that delay discounting is a less influential predictor of alcohol use than widely assumed in the literature. Our results show quite robustly that the effect of delay discounting is indeed very small, but not completely absent. How relevant this effect is in practice remains open. Another aspect of the debate surrounding DRD is its interpretation as a measure of impulsivity. DRD rates have shown surprisingly low associations with other measures of impulsivity (Stahl et al., 2014; Strickland & Johnson, 2021), including the present study. Therefore, we agree that the term impulsivity should be avoided when interpreting DRD rates. Conversely, an exploratory model revealed that DRD rates did not appear to contribute unique variance in AUDIT scores on top of scores of the Barratt Impulsiveness Scale (Data S3.7). It could therefore be speculated whether the small association between DRD rates and alcohol use reflects the limited shared variance between DRD and impulsivity. However, self‐report measures, such as BIS‐15 and AUDIT, usually correlate higher with each other than with behavioral measures, such as DRD (Dang et al., 2020). This might explain why DRD did not explain incremental variance after controlling for the relatively large association between impulsivity and alcohol use observed in our study (*r* = 0.36).

### Strengths and limitations

Using an online experiment, we collected a relatively large sample compared to the literature on clinical and subclinical populations. Although the sample size was sufficient to detect small main effects, the shared variance between DRD and third variables, such as education and age, further reduces DRD's unique effects, indicating that larger samples are needed for reliable estimation. The sample can be described as moderate to heavy drinkers with weekly drinking quantities (mean 24.43 alcohol units per week or 27.92 g/day) and AUDIT scores (mean 11.76) above commonly reported thresholds for risky drinking (AUDIT >10, weekly consumption of >14 units). Female participants averaged 20.80 alcohol units per week (or 23.8 g/day), exceeding the WHO threshold of 20 g/day for low‐risk drinking in women. In contrast, male participants consumed 28.1 alcohol units per week (or 32.1 g/day), below the WHO threshold of 40 g/day for low‐risk drinking in men. Figure 1B reveals a pattern of weekend drinking for most participants. Our data, lacking diagnostic criteria, allow conclusions about the association between delay discounting and alcohol use rather than addiction. However, by prefiltering participants with a minimum number of 10 alcohol units per week, we were able to achieve a broad distribution of low to high AUD risk without floor effects typically observed in general population samples (Figure 1A). This allowed us to dimensionally assess the effects of DRD and SES in a subclinical sample at risk for developing AUD. This has three major advantages: if DRD is indeed a risk factor for the development of AUD (Bernhardt et al., 2017; Dougherty et al., 2015; Fröhner et al., 2022), their association should not only be visible in dichotomous case‐control studies but also in a dimensional sample. In addition, more severe AUD levels (as observed in patient samples) are more likely to confound measures such as income due to the effects of AUD on socioeconomic status. Lastly, many DRD studies are confined to heavily affected patient groups, despite only a minority of individuals with alcohol use disorder seeking treatment (Mekonen et al., 2021).

Importantly, we were able to sample a distribution of income closely matching UK population levels (HM Revenue and Customs, 2022). However, the study sample is biased towards highly educated participants, with 61.3% of participants having at least a Bachelor's degree, compared to 42.2% in the general UK population (OECD, 2022). This possibly reduced our ability to detect strong socioeconomic effects. It is important to note that online platforms like Prolific may undersample individuals with low socioeconomic status or severe addiction. Therefore, online samples are not ideal for evaluating the role of socioeconomic factors in addiction. Additionally, our sampling method biased the study population towards higher‐than‐average alcohol use (compared to the UK average) and an overrepresentation of women (relative to their proportion among frequent drinkers). This limits the generalizability of our findings.

Samples from online studies are subject to criticism regarding their data quality (Peer et al., 2022). We restricted our sample to experienced respondents with high approval ratings and manually checked the data quality by examining response times and decision patterns. Based on the preregistered exclusion criteria, only one respondent had to be excluded. Importantly, the present discounting paradigm was developed and validated on Prolific subjects. Our previous studies with Prolific participants used an extended version of the current discounting task with out‐of‐sample cross‐validation, generating individualized trials based on model‐predicted choice behavior, and showed excellent predictive accuracy using the same quality checks (Thome, Pinger, Durstewitz, et al., 2022; Thome, Pinger, Halli, et al., 2022). We are therefore confident of the data quality in the present study. However, similar to psychology students, experienced study participants may conduct studies with increasing knowledge and routine, limiting the external validity of our findings. Lastly, the absence of longitudinal data limits our ability to establish causal relationships. Taken together, our study allows for the exploration of DRD's influence on high‐functioning heavy drinkers, contributing significantly to the field.

## CONCLUSIONS

We found that DRD remains a significant predictor of alcohol use, albeit with a small effect size when accounting for socioeconomic factors including income, education, and subjective SES. These findings support the robustness of the association between DRD and risky alcohol use commonly observed in prior studies. However, exploratory analyses revealed a potential confounding effect of age and education, which needs to be confirmed in future studies. As the incremental effect of DRD after controlling for SES is very small, small methodological changes (e.g., number of covariates and sample size) can heavily influence whether a significant effect of DRD can be detected. Therefore, we suggest that socioeconomic and demographic variables should systematically be accounted for in future studies investigating DRD and alcohol use, including longitudinal studies.

Lastly, we found no significant association between DLD and alcohol use. This finding was independent of subgroups, for example, low‐discounters and individuals at high/low risk for AUD. Monetary aversion discounting may therefore not be a useful task in addiction research.

## FUNDING INFORMATION

This study was supported by the German Research Foundation (DFG) within the collaborative research center TRR 265, subproject B08, granted to GK, PK, and WS.

## CONFLICT OF INTEREST STATEMENT

The authors declare that the research was conducted in the absence of any commercial or financial relationships that could be construed as a potential conflict of interest.

## Supporting information


Data S1.

Data S2.

## Data Availability

Raw data, analysis scripts, and a codebook of all variables are publicly available at https://osf.io/85k3h/. All code needed for the setup and execution of the online study is available at https://github.com/MathieuPinger/discounting‐online/tree/main/Discounting_AUD_Socioeconomic.

## References

[acer15469-bib-0001] Acheson, A. , Vincent, A.S. , Cohoon, A. & Lovallo, W.R. (2019) Early life adversity and increased delay discounting: findings from the family health patterns project. Experimental and Clinical Psychopharmacology, 27(2), 153–159. Available from: 10.1037/pha0000241 30556730 PMC6719544

[acer15469-bib-0002] Adler, N.E. , Epel, E.S. , Castellazzo, G. & Ickovics, J.R. (2000) Relationship of subjective and objective social status with psychological and physiological functioning: preliminary data in healthy, white women. Health Psychology, 19(6), 586–592. Available from: 10.1037/0278-6133.19.6.586 11129362

[acer15469-bib-0003] Agabio, R. , Pisanu, C. , Gessa, G.L. & Franconi, F. (2017) Sex differences in alcohol use disorder. Current Medicinal Chemistry, 24(24), 2661–2670. Available from: 10.2174/0929867323666161202092908 27915987

[acer15469-bib-0004] Ahn, W.‐Y. , Gu, H. , Shen, Y. , Haines, N. , Hahn, H.A. , Teater, J.E. et al. (2020) Rapid, precise, and reliable measurement of delay discounting using a Bayesian learning algorithm. Scientific Reports, 10(1), 12091. Available from: 10.1038/s41598-020-68587-x 32694654 PMC7374100

[acer15469-bib-0005] Alf, E.F., Jr. & Graf, R.G. (1999) Asymptotic confidence limits for the difference between two squared multiple correlations: a simplified approach. Psychological Methods, 4(1), 70–75. Available from: 10.1037/1082-989X.4.1.70

[acer15469-bib-0006] Amlung, M. & MacKillop, J. (2014) Clarifying the relationship between impulsive delay discounting and nicotine dependence. Psychology of Addictive Behaviors: Journal of the Society of Psychologists in Addictive Behaviors, 28(3), 761–768. Available from: 10.1037/a0036726 24841186 PMC4165767

[acer15469-bib-0007] Amlung, M. , Vedelago, L. , Acker, J. , Balodis, I. & MacKillop, J. (2017) Steep delay discounting and addictive behavior: a meta‐analysis of continuous associations. Addiction, 112(1), 51–62. Available from: 10.1111/add.13535 PMC5148639 27450931

[acer15469-bib-0008] Anokhin, A.P. , Golosheykin, S. , Grant, J.D. & Heath, A.C. (2011) Heritability of delay discounting in adolescence: a longitudinal twin study. Behavior Genetics, 41(2), 175–183. Available from: 10.1007/s10519-010-9384-7 20700643 PMC3036802

[acer15469-bib-0009] Bailey, A.J. , Gerst, K. & Finn, P.R. (2018) Delay discounting of losses and rewards in alcohol use disorder: the effect of working memory load. Psychology of Addictive Behaviors: Journal of the Society of Psychologists in Addictive Behaviors, 32(2), 197–204. Available from: 10.1037/adb0000341 29355332 PMC5858963

[acer15469-bib-0010] Bailey, A.J. , Romeu, R.J. & Finn, P.R. (2021) The problems with delay discounting: a critical review of current practices and clinical applications. Psychological Medicine, 51(11), 1799–1806. Available from: 10.1017/S0033291721002282 34184631 PMC8381235

[acer15469-bib-0011] Beard, E. , Brown, J. , West, R. , Kaner, E. , Meier, P. & Michie, S. (2019) Associations between socio‐economic factors and alcohol consumption: a population survey of adults in England. PLoS One, 14(2), e0209442. Available from: 10.1371/journal.pone.0209442 30716098 PMC6361426

[acer15469-bib-0012] Becker, G.S. & Mulligan, C.B. (1997) The endogenous determination of time preference. The Quarterly Journal of Economics, 112(3), 729–758. Available from: 10.1162/003355397555334

[acer15469-bib-0013] Bernhardt, N. , Nebe, S. , Pooseh, S. , Sebold, M. , Sommer, C. , Birkenstock, J. et al. (2017) Impulsive decision making in young adult social drinkers and detoxified alcohol‐dependent patients: a cross‐sectional and longitudinal study. Alcoholism, Clinical and Experimental Research, 41(10), 1794–1807. Available from: 10.1111/acer.13481 28815629

[acer15469-bib-0014] Bickel, W.K. , Johnson, M.W. , Koffarnus, M.N. , MacKillop, J. & Murphy, J.G. (2014) The behavioral economics of substance use disorders: reinforcement pathologies and their repair. Annual Review of Clinical Psychology, 10(1), 641–677. Available from: 10.1146/annurev-clinpsy-032813-153724 PMC4501268 24679180

[acer15469-bib-0015] Bickel, W.K. , Wilson, A.G. , Chen, C. , Koffarnus, M.N. & Franck, C.T. (2016) Stuck in time: negative income shock constricts the temporal window of valuation spanning the future and the past. PLoS One, 11(9), e0163051. Available from: 10.1371/journal.pone.0163051 27631760 PMC5025165

[acer15469-bib-0016] Collins, R.L. , Parks, G.A. & Marlatt, G.A. (1985) Social determinants of alcohol consumption: the effects of social interaction and model status on the self‐administration of alcohol. Journal of Consulting and Clinical Psychology, 53(2), 189–200. Available from: 10.1037/0022-006X.53.2.189 3998247

[acer15469-bib-0017] Cumming, G. & Finch, S. (2005) Inference by eye: confidence intervals and how to read pictures of data. American Psychologist, 60(2), 170–180. Available from: 10.1037/0003-066X.60.2.170 15740449

[acer15469-bib-0018] Dang, J. , Liu, X. , Xiao, S. , Mao, L. , Chan, K.T. , Li, C. et al. (2020) The beauty of the zero: replications and extensions of the hidden‐zero effect in delay discounting tasks. Social Psychological and Personality Science, 12, 194855062092945. Available from: 10.1177/1948550620929454

[acer15469-bib-0019] de Leeuw, J.R. (2015) jsPsych: a JavaScript library for creating behavioral experiments in a web browser. Behavior Research Methods, 47(1), 1–12. Available from: 10.3758/s13428-014-0458-y 24683129

[acer15469-bib-0020] de Wit, H. , Flory, J.D. , Acheson, A. , McCloskey, M. & Manuck, S.B. (2007) IQ and nonplanning impulsivity are independently associated with delay discounting in middle‐aged adults. Personality and Individual Differences, 42(1), 111–121. Available from: 10.1016/j.paid.2006.06.026

[acer15469-bib-0021] DeHart, W.B. , Friedel, J.E. , Berry, M. , Frye, C.C.J. , Galizio, A. & Odum, A.L. (2020) Comparison of delay discounting of different outcomes in cigarette smokers, smokeless tobacco users, e‐cigarette users, and non‐tobacco users. Journal of the Experimental Analysis of Behavior, 114(2), 203–215. Available from: 10.1002/jeab.623 32852106 PMC8269745

[acer15469-bib-0022] Diemer, M.A. , Mistry, R.S. , Wadsworth, M.E. , López, I. & Reimers, F. (2013) Best practices in conceptualizing and measuring social class in psychological research. Analyses of Social Issues and Public Policy, 13(1), 77–113. Available from: 10.1111/asap.12001

[acer15469-bib-0023] Doidge, J.L. , Flora, D.B. & Toplak, M.E. (2021) A meta‐analytic review of sex differences on delay of gratification and temporal discounting tasks in ADHD and typically developing samples. Journal of Attention Disorders, 25(4), 540–561. Available from: 10.1177/1087054718815588 30596297

[acer15469-bib-0024] Dougherty, D.M. , Lake, S.L. , Mathias, C.W. , Ryan, S.R. , Bray, B.C. , Charles, N.E. et al. (2015) Behavioral impulsivity and risk‐taking trajectories across early adolescence in youths with and without family histories of alcohol and other drug use disorders. Alcoholism, Clinical and Experimental Research, 39(8), 1501–1509. Available from: 10.1111/acer.12787 26173617 PMC4630668

[acer15469-bib-0025] Exum, A.C. , Sutton, C.A. , Bellitti, J.S. , Yi, R. & Fazzino, T.L. (2023) Delay discounting and substance use treatment outcomes: a systematic review focused on treatment outcomes and discounting methodology. Journal of Substance Use and Addiction Treatment, 149, 209037. Available from: 10.1016/j.josat.2023.209037 37072099 PMC10429418

[acer15469-bib-0026] Fröhner, J.H. , Ripke, S. , Jurk, S. , Li, S.‐C. , Banaschewski, T. , Bokde, A.L.W. et al. (2022) Associations of delay discounting and drinking trajectories from ages 14 to 22. Alcoholism, Clinical and Experimental Research, 46(4), 667–681. Available from: 10.1111/acer.14799 35257381 PMC9018624

[acer15469-bib-0027] Garza, J.R. , Glenn, B.A. , Mistry, R.S. , Ponce, N.A. & Zimmerman, F.J. (2017) Subjective social status and self‐reported health among US‐born and immigrant Latinos. Journal of Immigrant and Minority Health, 19(1), 108–119. Available from: 10.1007/s10903-016-0346-x 26895151

[acer15469-bib-0028] Garzón, B. , Kurth‐Nelson, Z. , Bäckman, L. , Nyberg, L. & Guitart‐Masip, M. (2022) Investigating associations of delay discounting with brain structure, working memory, and episodic memory. Cerebral Cortex, 33(5), 1669–1678. Available from: 10.1093/cercor/bhac164 PMC9977379 35488441

[acer15469-bib-0029] Gerst, K.R. , Gunn, R.L. & Finn, P.R. (2017) Delay discounting of losses in alcohol use disorders and antisocial psychopathology: effects of a working memory load. Alcoholism: Clinical and Experimental Research, 41(10), 1768–1774. Available from: 10.1111/acer.13472 28792623 PMC5626637

[acer15469-bib-0030] Grant, B.F. , Goldstein, R.B. , Saha, T.D. , Chou, S.P. , Jung, J. , Zhang, H. et al. (2015) Epidemiology of DSM‐5 alcohol use disorder: results from the National Epidemiologic Survey on alcohol and related conditions III. JAMA Psychiatry, 72(8), 757–766. Available from: 10.1001/jamapsychiatry.2015.0584 26039070 PMC5240584

[acer15469-bib-0031] Green, L. , Myerson, J. , Lichtman, D. , Rosen, S. & Fry, A. (1996) Temporal discounting in choice between delayed rewards: the role of age and income. Psychology and Aging, 11(1), 79–84. Available from: 10.1037//0882-7974.11.1.79 8726373

[acer15469-bib-0032] Green, L. , Myerson, J. , Oliveira, L. & Chang, S.E. (2014) Discounting of delayed and probabilistic losses over a wide range of amounts. Journal of the Experimental Analysis of Behavior, 101(2), 186–200. Available from: 10.1901/jeab.2014.101-186 24745086 PMC4056767

[acer15469-bib-0033] Hardaway, C.R. & Cornelius, M.D. (2014) Economic hardship and adolescent problem drinking: family processes as mediating influences. Journal of Youth and Adolescence, 43(7), 1191–1202. Available from: 10.1007/s10964-013-0063-x 24248327 PMC5484145

[acer15469-bib-0034] HM Revenue and Customs . (2022) Personal incomes statistics 2021 to 2022: commentary. GOV.UK. https://www.gov.uk/government/statistics/personal‐incomes‐statistics‐for‐the‐tax‐year‐2021‐to‐2022/personal‐incomes‐statistics‐2021‐to‐2022‐commentary.

[acer15469-bib-0035] Ishii, K. (2015) Subjective socioeconomic status and cigarette smoking interact to delay discounting. Springerplus, 4(1), 560. Available from: 10.1186/s40064-015-1361-4 26435906 PMC4586184

[acer15469-bib-0036] Ishii, K. , Eisen, C. & Hitokoto, H. (2017) The effects of social status and culture on delay discounting. Japanese Psychological Research, 59(3), 230–237. Available from: 10.1111/jpr.12154

[acer15469-bib-0037] Jaroni, J.L. , Wright, S.M. , Lerman, C. & Epstein, L.H. (2004) Relationship between education and delay discounting in smokers. Addictive Behaviors, 29(6), 1171–1175. Available from: 10.1016/j.addbeh.2004.03.014 15236819

[acer15469-bib-0038] Jenkins, R. , Bhugra, D. , Bebbington, P. , Brugha, T. , Farrell, M. , Coid, J. et al. (2008) Debt, income and mental disorder in the general population. Psychological Medicine, 38(10), 1485–1493. Available from: 10.1017/S0033291707002516 18184442

[acer15469-bib-0039] MacKillop, J. , Amlung, M.T. , Few, L.R. , Ray, L.A. , Sweet, L.H. & Munafò, M.R. (2011) Delayed reward discounting and addictive behavior: a meta‐analysis. Psychopharmacology, 216(3), 305–321. Available from: 10.1007/s00213-011-2229-0 21373791 PMC3201846

[acer15469-bib-0040] Martínez‐Loredo, V. (2023) Critical appraisal of the discussion on delay discounting by Bailey et al. and Stein et al.: a scientific proposal for a reinforcer pathology theory 3.0. New Ideas in Psychology, 69, 101006. Available from: 10.1016/j.newideapsych.2022.101006

[acer15469-bib-0041] Mazur, J.E. (1987) An adjusting procedure for studying delayed reinforcement. In: Commons, M.L. , Mazur, J.E. , Nevin, J.A. , & Rachlin, H. (Eds.)The effect of delay and of intervening events on reinforcement value. Hillsdale, NJ: Lawrence Erlbaum Associates, Inc, pp. 55–73.

[acer15469-bib-0042] Mekonen, T. , Chan, G.C.K. , Connor, J. , Hall, W. , Hides, L. & Leung, J. (2021) Treatment rates for alcohol use disorders: a systematic review and meta‐analysis. Addiction, 116(10), 2617–2634. Available from: 10.1111/add.15357 33245581

[acer15469-bib-0043] Mellis, A.M. , Athamneh, L.N. , Stein, J.S. , Sze, Y.Y. , Epstein, L.H. & Bickel, W.K. (2018) Less is more: negative income shock increases immediate preference in cross commodity discounting and food demand. Appetite, 129, 155–161. Available from: 10.1016/j.appet.2018.06.032 29959952 PMC6156798

[acer15469-bib-0044] Mitchell, J.M. , Fields, H.L. , D'Esposito, M. & Boettiger, C.A. (2005) Impulsive responding in alcoholics. Alcoholism: Clinical and Experimental Research, 29(12), 2158–2169. Available from: 10.1097/01.alc.0000191755.63639.4a 16385186

[acer15469-bib-0045] Myerson, J. , Baumann, A.A. & Green, L. (2017) Individual differences in delay discounting: differences are quantitative with gains, but qualitative with losses. Journal of Behavioral Decision Making, 30(2), 359–372. Available from: 10.1002/bdm.1947

[acer15469-bib-0046] Najdzionek, P. , McIntyre‐Wood, C. , Amlung, M. & MacKillop, J. (2023) Incorporating socioeconomic status into studies on delay discounting and health via subjective financial status: an initial validation in tobacco use. Experimental and Clinical Psychopharmacology, 31(2), 475–481. Available from: 10.1037/pha0000628 36595454

[acer15469-bib-0047] OECD . (2022) Educational attainment and labour‐force status . https://stats.oecd.org/Index.aspx?DataSetCode=EAG_NEAC

[acer15469-bib-0048] Peer, E. , Rothschild, D. , Gordon, A. , Evernden, Z. & Damer, E. (2022) Data quality of platforms and panels for online behavioral research. Behavior Research Methods, 54(4), 1643–1662. Available from: 10.3758/s13428-021-01694-3 34590289 PMC8480459

[acer15469-bib-0049] R Core Team . (2022) R: a language and environment for statistical computing [Software]. Vienna, Austria: R Foundation for Statistical Computing. https://www.R‐project.org/

[acer15469-bib-0050] Rachlin, H. (2006) Notes on discounting. Journal of the Experimental Analysis of Behavior, 85(3), 425–435. Available from: 10.1901/jeab.2006.85-05 16776060 PMC1459845

[acer15469-bib-0051] Rachlin, H. , Raineri, A. & Cross, D. (1991) Subjective probability and delay. Journal of the Experimental Analysis of Behavior, 55(2), 233–244. Available from: 10.1901/jeab.1991.55-233 2037827 PMC1323057

[acer15469-bib-0052] Reimers, S. , Maylor, E.A. , Stewart, N. & Chater, N. (2009) Associations between a one‐shot delay discounting measure and age, income, education and real‐world impulsive behavior. Personality and Individual Differences, 47(8), 973–978. Available from: 10.1016/j.paid.2009.07.026

[acer15469-bib-0053] Ruggeri, K. , Ashcroft‐Jones, S. , Abate Romero Landini, G. , Al‐Zahli, N. , Alexander, N. , Andersen, M.H. et al. (2023) The persistence of cognitive biases in financial decisions across economic groups. Scientific Reports, 13(1), 10329. Available from: 10.1038/s41598-023-36339-2 37365245 PMC10293260

[acer15469-bib-0054] Ruggeri, K. , Panin, A. , Vdovic, M. , Većkalov, B. , Abdul‐Salaam, N. , Achterberg, J. et al. (2022) The globalizability of temporal discounting. Nature Human Behaviour, 6(10), 1386–1397. Available from: 10.1038/s41562-022-01392-w PMC9584811 35817934

[acer15469-bib-0055] Saunders, J.B. , Aasland, O.G. , Babor, T.F. , de la Fuente, J.R. & Grant, M. (1993) Development of the alcohol use disorders identification test (AUDIT): WHO collaborative project on early detection of persons with harmful alcohol consumption—II. Addiction, 88(6), 791–804. Available from: 10.1111/j.1360-0443.1993.tb02093.x 8329970

[acer15469-bib-0056] Schneider, S.L. (2013) The international standard classification of education 2011. In: Elisabeth Birkelund, G. (Ed.) Class and stratification analysis, Vol. Bd. 30. Leeds, UK: Emerald Group Publishing Limited, pp. 365–379. Available from: 10.1108/S0195-6310(2013)0000030017

[acer15469-bib-0057] Seaman, K.L. , Abiodun, S. , Fenn, Z. , Samanez‐Larkin, G. & Mata, R. (2020) Temporal discounting across adulthood: a systematic review and meta‐analysis . PsyArXiv 10.31234/osf.io/7ysxa PMC8827494 35113618

[acer15469-bib-0058] Shuai, R. , Anker, J.J. , Bravo, A.J. , Kushner, M.G. & Hogarth, L. (2022) Risk pathways contributing to the alcohol harm paradox: socioeconomic deprivation confers susceptibility to alcohol dependence via greater exposure to aversive experience, internalizing symptoms and drinking to cope. Frontiers in Behavioral Neuroscience, 16, 821693. Available from: 10.3389/fnbeh.2022.821693 35237137 PMC8883115

[acer15469-bib-0059] Spinella, M. (2007) Normative data and a short form of the Barratt impulsiveness scale. The International Journal of Neuroscience, 117(3), 359–368. Available from: 10.1080/00207450600588881 17365120

[acer15469-bib-0060] Stahl, C. , Voss, A. , Schmitz, F. , Nuszbaum, M. , Tuscher, O. , Lieb, K. et al. (2014) Behavioral components of impulsivity. Journal of Experimental Psychology: General, 143(2), 850–886. Available from: 10.1037/a0033981 23957282

[acer15469-bib-0061] Stanley, D. (2015) *apaTables: Create American Psychological Association (APA) Style Tables* (S. 2.0.8) [Dataset]. 10.32614/CRAN.package.apaTables

[acer15469-bib-0062] Stein, J.S. , MacKillop, J. , McClure, S.M. & Bickel, W.K. (2023) Unsparing self‐critique strengthens the field, but Bailey et al. Overstate the ‘problems with delay discounting’. Psychological Medicine, 53(4), 1658–1659. Available from: 10.1017/S0033291721005286 35225188 PMC10009363

[acer15469-bib-0063] Story, G. , Vlaev, I. , Seymour, B. , Darzi, A. & Dolan, R. (2014) Does temporal discounting explain unhealthy behavior? A systematic review and reinforcement learning perspective. Frontiers in Behavioral Neuroscience, 8, 76. Available from: 10.3389/fnbeh.2014.00076 24659960 PMC3950931

[acer15469-bib-0064] Strickland, J.C. & Johnson, M.W. (2021) Rejecting impulsivity as a psychological construct: a theoretical, empirical, and sociocultural argument. Psychological Review, 128(2), 336–361. Available from: 10.1037/rev0000263 32969672 PMC8610097

[acer15469-bib-0071] Sutton, R.S. & Barto, A.G. (2018). Reinforcement Learning: an Introduction. Cambridge, MA: MIT Press.

[acer15469-bib-0065] Takahashi, T. , Ohmura, Y. , Oono, H. & Radford, M. (2009) Alcohol use and discounting of delayed and probabilistic gain and loss. Neuro Endocrinology Letters, 30(6), 749–752. 20038936

[acer15469-bib-0066] The Department of Health and Social Care . (2020) Alcohol use screening tests. GOV.UK. https://www.gov.uk/government/publications/alcohol‐use‐screening‐tests

[acer15469-bib-0067] Thome, J. , Pinger, M. , Durstewitz, D. , Sommer, W.H. , Kirsch, P. & Koppe, G. (2022) Model‐based experimental manipulation of probabilistic behavior in interpretable behavioral latent variable models. Frontiers in Neuroscience, 16, 1077735. Available from: 10.3389/fnins.2022.1077735 36699538 PMC9868576

[acer15469-bib-0068] Thome, J. , Pinger, M. , Halli, P. , Durstewitz, D. , Sommer, W.H. , Kirsch, P. et al. (2022) A model guided approach to evoke homogeneous behavior during temporal reward and loss discounting. Frontiers in Psychiatry, 13, 846119. Available from: 10.3389/fpsyt.2022.846119 35800024 PMC9253427

[acer15469-bib-0069] Tunney, R.J. (2022) Economic and social deprivation predicts impulsive choice in children. Scientific Reports, 12(1), 8942. Available from: 10.1038/s41598-022-12872-4 35624120 PMC9142580

[acer15469-bib-0070] Watts, T.W. , Duncan, G.J. & Quan, H. (2018) Revisiting the marshmallow test: a conceptual replication investigating links between early delay of gratification and later outcomes. Psychological Science, 29(7), 1159–1177. Available from: 10.1177/0956797618761661 29799765 PMC6050075

