# Supplementary material for: Delay discounting of rewards and losses, alcohol use, and the influence of socioeconomic factors: A cross‐sectional online study in frequent drinkers
Source: Alcohol Clin Exp Res (Hoboken). 2024 Oct 22;48(12):2364–77. doi: 10.1111/acer.15469 (PMC11629459; doi:10.1111/acer.15469)
Supplement: Supplementary file 1 — Data S1. [file ACER-48-2364-s002.docx]

**Supplementary Data**

**Supplementary Data S1: Description of Discounting Indices (confirmatory and exploratory)**

1. Hypothesis testing was preregistered using natural log-transformed κ parameters obtained from the hyperboloid model (see above) after adding a constant of 0.0001 to account for zero-values. Log(κ) is the most common index of discounting and determines the steepness of the devaluation of future rewards/losses according to a hyperboloid curve. Higher log(κ) values indicate steeper discounting Log(κ_R_) is used to refer to DRD, log(κ_L_) is used to refer to DLD.
2. The discounting factor $df:= \frac{1}{1+\kappa{\cdot D}^{s}}$ ∈ [0, 1] at delay $D=365$ days was used as an exploratory measure. The discounting factor includes both the discounting parameter κ and the temporal scaling parameter s and yields the subjective value for any outcome for a specific delay when multiplied by its objective value. For instance, if $df$ = 0.5 at D = 365 days, the subjective value of £100 is £50. Lower discounting factors indicate steeper discounting.
3. Frequency of discounted choices relative to all completed trials was used as a secondexploratory behavioral measure. Discounted choices were defined as immediate choices in the reward condition and delayed choices in the loss condition. In contrast to log(κ ) and the discounting factor, discounting frequency is an atheoretical quantification of how often participants chose the immediate/delayed options. As discounting models aim to predict choice behavior, we included this measure as a sanity check: vastly different results in analyses using model parameters (e.g. log(κ)) and discounting frequencies could indicate problems with the validity of the behavioral models.

**Supplementary Data S2**

Means, standard deviations, and correlations of all variables. For display purposes, the table has been outsourced as an .xlxs file.

**Supplementary Data S3.1**

*Regression results using AUDIT as the criterion and Education and the Reward Discounting Factor as predictors*

| Predictor | *b* | *b*  95% CI  [LL, UL] | *β* | *β*  95% CI  [LL, UL] | *sr^2^* | *sr^2^*  95% CI  [LL, UL] | *r* | Fit | Difference |
| --- | --- | --- | --- | --- | --- | --- | --- | --- | --- |
| (Intercept) | 14.31** | [12.43, 16.18] |  |  |  |  |  |  |  |
| Education | -0.59** | [-1.00, -0.18] | -0.15 | [-0.26, -0.05] | .02 | [.00, .06] | -.15** |  |  |
|  |  |  |  |  |  |  |  | *R^2^*  = .023** |  |
|  |  |  |  |  |  |  |  | 95% CI[.00,.06] |  |
|  |  |  |  |  |  |  |  |  |  |
| (Intercept) | 15.34** | [13.24, 17.44] |  |  |  |  |  |  |  |
| Education | -0.53* | [-0.94, -0.12] | -0.14 | [-0.24, -0.03] | .02 | [-.01, .05] | -.15** |  |  |
| Reward Discounting Factor | -2.24* | [-4.33, -0.14] | -0.11 | [-0.22, -0.01] | .01 | [-.01, .04] | -.13* |  |  |
|  |  |  |  |  |  |  |  | *R^2^*  = .036** | Δ*R^2^*  = .013* |
|  |  |  |  |  |  |  |  | 95% CI[.01,.08] | 95% CI[-.01, .04] |
|  |  |  |  |  |  |  |  |  |  |

*Note.* *b* represents unstandardized regression weights. *β* indicates the standardized regression weights. *sr^2^* represents the semi-partial correlation squared. *r* represents the zero-order correlation. *LL* and *UL* indicate the lower and upper limits of a confidence interval, respectively.
* indicates *p* < .05. ** indicates *p* < .01.

**Supplementary Data S3.2**

*Regression results using AUDIT as the criterion and Education and Relative Frequency of Reward Discounting as predictors*

| Predictor | *b* | *b*  95% CI  [LL, UL] | *β* | *β*  95% CI  [LL, UL] | *sr^2^* | *sr^2^*  95% CI  [LL, UL] | *r* | Fit | Difference |
| --- | --- | --- | --- | --- | --- | --- | --- | --- | --- |
| (Intercept) | 14.31** | [12.43, 16.18] |  |  |  |  |  |  |  |
| Education | -0.59** | [-1.00, -0.18] | -0.15 | [-0.26, -0.05] | .02 | [.00, .06] | -.15** |  |  |
|  |  |  |  |  |  |  |  | *R^2^*  = .023** |  |
|  |  |  |  |  |  |  |  | 95% CI[.00,.06] |  |
|  |  |  |  |  |  |  |  |  |  |
| (Intercept) | 12.18** | [9.48, 14.89] |  |  |  |  |  |  |  |
| Education | -0.51* | [-0.92, -0.10] | -0.13 | [-0.24, -0.02] | .02 | [-.01, .04] | -.15** |  |  |
| Rel. Frequency of Reward Discounting | 0.04* | [0.00, 0.07] | 0.12 | [0.01, 0.22] | .01 | [-.01, .04] | .14** |  |  |
|  |  |  |  |  |  |  |  | *R^2^*  = .036** | Δ*R^2^*  = .013* |
|  |  |  |  |  |  |  |  | 95% CI[.01,.08] | 95% CI[-.01, .04] |
|  |  |  |  |  |  |  |  |  |  |

*Note.* *b* represents unstandardized regression weights. *β* indicates the standardized regression weights. *sr^2^* represents the semi-partial correlation squared. *r* represents the zero-order correlation. *LL* and *UL* indicate the lower and upper limits of a confidence interval, respectively.
* indicates *p* < .05. ** indicates *p* < .01.

**Supplementary Data S3.3**

*Regression results using AUDIT as the criterion and Education, Subjective Socioeconomic Status (SSS) and log(kR) as predictors*

| Predictor | *b* | *b*  95% CI  [LL, UL] | *β* | *β*  95% CI  [LL, UL] | *sr^2^* | *sr^2^*  95% CI  [LL, UL] | *r* | Fit | Difference |
| --- | --- | --- | --- | --- | --- | --- | --- | --- | --- |
| (Intercept) | 15.88** | [13.35, 18.41] |  |  |  |  |  |  |  |
| Education | -0.47* | [-0.90, -0.04] | -0.12 | [-0.23, -0.01] | .01 | [-.01, .04] | -.15** |  |  |
| SSS | -0.38 | [-0.79, 0.03] | -0.10 | [-0.21, 0.01] | .01 | [-.01, .03] | -.14* |  |  |
|  |  |  |  |  |  |  |  | *R^2^*  = .033** |  |
|  |  |  |  |  |  |  |  | 95% CI[.00,.07] |  |
|  |  |  |  |  |  |  |  |  |  |
| (Intercept) | 15.97** | [13.45, 18.49] |  |  |  |  |  |  |  |
| Education | -0.41 | [-0.84, 0.02] | -0.11 | [-0.22, 0.01] | .01 | [-.01, .03] | -.15** |  |  |
| SSS | -0.32 | [-0.73, 0.10] | -0.09 | [-0.20, 0.03] | .01 | [-.01, .02] | -.14* |  |  |
| log(kR) | 0.20* | [0.01, 0.40] | 0.11 | [0.01, 0.22] | .01 | [-.01, .04] | .15** |  |  |
|  |  |  |  |  |  |  |  | *R^2^*  = .045** | Δ*R^2^*  = .012* |
|  |  |  |  |  |  |  |  | 95% CI[.01,.09] | 95% CI[-.01, .04] |
|  |  |  |  |  |  |  |  |  |  |

*Note.* SSS = Subjective Socioeconomic Status. *b* represents unstandardized regression weights. *β* indicates the standardized regression weights. *sr^2^* represents the semi-partial correlation squared. *r* represents the zero-order correlation. *LL* and *UL* indicate the lower and upper limits of a confidence interval, respectively.
* indicates *p* < .05. ** indicates *p* < .01.

SSS = Subjective Socioeconomic Status

**Supplementary Data S3.4**

*Regression results using DDQ scores (= average weekly 8g alcohol units over the last 3 months) as the criterion and Education and log(kR) as predictors*

| Predictor | *b* | *b*  95% CI  [LL, UL] | *β* | *β*  95% CI  [LL, UL] | *sr^2^* | *sr^2^*  95% CI  [LL, UL] | *r* | Fit | Difference |
| --- | --- | --- | --- | --- | --- | --- | --- | --- | --- |
| (Intercept) | 29.36** | [23.61, 35.12] |  |  |  |  |  |  |  |
| Education | -1.14 | [-2.40, 0.11] | -0.10 | [-0.20, 0.01] | .01 | [.00, .04] | -.10 |  |  |
|  |  |  |  |  |  |  |  | *R^2^*  = .009 |  |
|  |  |  |  |  |  |  |  | 95% CI[.00,.04] |  |
|  |  |  |  |  |  |  |  |  |  |
| (Intercept) | 30.36** | [24.55, 36.18] |  |  |  |  |  |  |  |
| Education | -0.91 | [-2.18, 0.37] | -0.08 | [-0.18, 0.03] | .01 | [-.01, .02] | -.10 |  |  |
| log(kR) | 0.59* | [0.00, 1.17] | 0.11 | [0.00, 0.22] | .01 | [-.01, .03] | .12* |  |  |
|  |  |  |  |  |  |  |  | *R^2^*  = .021* | Δ*R^2^*  = .011* |
|  |  |  |  |  |  |  |  | 95% CI[.00,.06] | 95% CI[-.01, .03] |
|  |  |  |  |  |  |  |  |  |  |

*Note.* *b* represents unstandardized regression weights. *β* indicates the standardized regression weights. *sr^2^* represents the semi-partial correlation squared. *r* represents the zero-order correlation. *LL* and *UL* indicate the lower and upper limits of a confidence interval, respectively.
* indicates *p* < .05. ** indicates *p* < .01.

**Supplementary Data S3.5**

*Regression results using AUDIT scores as the criterion and Education, Age and log(kR) as predictors*

| Predictor | *b* | *b*  95% CI  [LL, UL] | *β* | *β*  95% CI  [LL, UL] | *sr^2^* | *sr^2^*  95% CI  [LL, UL] | *r* | Fit | Difference |
| --- | --- | --- | --- | --- | --- | --- | --- | --- | --- |
| (Intercept) | 19.17** | [16.24, 22.10] |  |  |  |  |  |  |  |
| Age | -0.11** | [-0.16, -0.06] | -0.22 | [-0.32, -0.12] | .05 | [.00, .09] | -.22** |  |  |
| Education | -0.59** | [-0.99, -0.19] | -0.15 | [-0.26, -0.05] | .02 | [-.01, .05] | -.15** |  |  |
|  |  |  |  |  |  |  |  | *R^2^*  = .071** |  |
|  |  |  |  |  |  |  |  | 95% CI[.03,.13] |  |
|  |  |  |  |  |  |  |  |  |  |
| (Intercept) | 19.06** | [16.13, 21.98] |  |  |  |  |  |  |  |
| Age | -0.10** | [-0.16, -0.05] | -0.20 | [-0.31, -0.10] | .04 | [-.00, .08] | -.22** |  |  |
| Education | -0.53* | [-0.94, -0.13] | -0.14 | [-0.24, -0.03] | .02 | [-.01, .05] | -.15** |  |  |
| log(kR) | 0.15 | [-0.04, 0.34] | 0.08 | [-0.02, 0.19] | .01 | [-.01, .02] | .15** |  |  |
|  |  |  |  |  |  |  |  | *R^2^*  = .078** | Δ*R^2^*  = .007 |
|  |  |  |  |  |  |  |  | 95% CI[.03,.13] | 95% CI[-.01, .02] |
|  |  |  |  |  |  |  |  |  |  |

*Note.* *b* represents unstandardized regression weights. *β* indicates the standardized regression weights. *sr^2^* represents the semi-partial correlation squared. *r* represents the zero-order correlation. *LL* and *UL* indicate the lower and upper limits of a confidence interval, respectively.
* indicates *p* < .05. ** indicates *p* < .01.

**Supplementary Data S3.6**

*Regression results using AUDIT scores as the criterion and log(kR), gender and their interaction as predictors*

| Predictor | *b* | *b*  95% CI  [LL, UL] | *sr^2^* | *sr^2^*  95% CI  [LL, UL] | Fit |
| --- | --- | --- | --- | --- | --- |
| (Intercept) | 11.26** | [9.95, 12.56] |  |  |  |
| Gender (0 = female, 1 = male) | 2.73** | [0.93, 4.53] | .03 | [-.01, .06] |  |
| log(kR) | 0.08 | [-0.19, 0.36] | .00 | [-.01, .01] |  |
| Gender*log(kR) | 0.34 | [-0.03, 0.71] | .01 | [-.01, .03] |  |
|  |  |  |  |  | *R^2^*  = .048** |
|  |  |  |  |  | 95% CI[.01,.09] |
|  |  |  |  |  |  |

*Note.* A significant *b*-weight indicates the semi-partial correlation is also significant. *b* represents unstandardized regression weights. *sr^2^* represents the semi-partial correlation squared. *LL* and *UL* indicate the lower and upper limits of a confidence interval, respectively.
* indicates p < .05. ** indicates p < .01.

**Supplementary Data S3.7**

*Regression results using AUDIT scores as the criterion and Barratt-Impulsiveness-Scale (BIS-15) scores and log(kR) as predictors*

| Predictor | *b* | *b*  95% CI  [LL, UL] | *β* | *β*  95% CI  [LL, UL] | *sr^2^* | *sr^2^*  95% CI  [LL, UL] | *r* | Fit | Difference |
| --- | --- | --- | --- | --- | --- | --- | --- | --- | --- |
| (Intercept) | 1.66 | [-1.19, 4.50] |  |  |  |  |  |  |  |
| BIS-15 | 0.33** | [0.24, 0.42] | 0.36 | [0.26, 0.46] | .13 | [.07, .20] | .36** |  |  |
|  |  |  |  |  |  |  |  | *R^2^*  = .131** |  |
|  |  |  |  |  |  |  |  | 95% CI[.07,.20] |  |
|  |  |  |  |  |  |  |  |  |  |
| (Intercept) | 2.65 | [-0.38, 5.69] |  |  |  |  |  |  |  |
| BIS-15 | 0.32** | [0.22, 0.41] | 0.35 | [0.25, 0.45] | .12 | [.05, .18] | .36** |  |  |
| log(kR) | 0.16 | [-0.01, 0.34] | 0.09 | [-0.01, 0.19] | .01 | [-.01, .03] | .15** |  |  |
|  |  |  |  |  |  |  |  | *R^2^*  = .139** | Δ*R^2^*  = .008 |
|  |  |  |  |  |  |  |  | 95% CI[.08,.20] | 95% CI[-.01, .03] |
|  |  |  |  |  |  |  |  |  |  |

*Note.* A significant *b*-weight indicates the β-weight and semi-partial correlation are also significant. *b* represents unstandardized regression weights. *β* indicates the standardized regression weights. *sr^2^* represents the semi-partial correlation squared. *r* represents the zero-order correlation. *LL* and *UL* indicate the lower and upper limits of a confidence interval, respectively.
* indicates *p* < .05. ** indicates *p* < .01.

| **Supplementary Data S4** | | | | | | |
| --- | --- | --- | --- | --- | --- | --- |
| *T-tests of selected variables between DLD non-discounters (n = 101, defined as <5% discounting) and DLD discounters (n = 239)* | | | | | | |
|  | Mean  (Non-Disc.) | Mean  (Disc.) | Difference | t | *df* | *p* |
| Age | 43.80 | 42.94 | 0.86 | 0.64 | 210.31 | .523 |
| AUDIT | 12.07 | 11.03 | 1.04 | 1.45 | 190.74 | .148 |
| BIS-15 | 30.83 | 30.25 | 0.58 | 0.75 | 194.91 | .456 |
| Education | 4.33 | 4.26 | 0.07 | 0.39 | 189.70 | .694 |
| Income Individual | 29,206.01 | 24,895.83 | 4,310.18 | 1.81 | 254.32 | .071 |
| *Note.* AUDIT = Alcohol Use Disorders Identification Test, BIS-15 = Barratt Impulsiveness Scale (Short Version) | | | | | | |

| **Supplementary Data S5** | | | | | | |
| --- | --- | --- | --- | --- | --- | --- |
| *T-tests of selected variables between male (n = 171) and female (n = 170) subgroups* | | | | | | |
|  | Mean  (Female) | Mean  (Male) | Difference | t | *df* | *p* |
| AUDIT | 10.96 | 12.56 | -1.59 | -2.44 | 338.76 | .015* |
| DDQ | 23.75 | 32.07 | -8.32 | -3.71 | 322.72 | < .001*** |
| BIS-15 | 30.42 | 30.88 | -0.46 | -0.64 | 327.66 | .524 |
| Education | 4.32 | 4.30 | 0.01 | 0.08 | 336.75 | .936 |
| Income Individual | 22,826.09 | 32,928.99 | -10,102.91 | -4.14 | 308.90 | < .001*** |
| log(κL) | -5.88 | -6.09 | 0.22 | 0.53 | 336.87 | .597 |
| log(κR) | -3.49 | -3.39 | -0.09 | -0.25 | 338.07 | .802 |
| *Note.* AUDIT = Alcohol Use Disorders Identification Test, DDQ = Daily Drinking Questionnaire, BIS-15 = Barratt Impulsiveness Scale (Short Version) | | | | | | |
